# Disruption of paternal circadian rhythm affects metabolic health in male offspring via nongerm cell factors

**DOI:** 10.1126/sciadv.abg6424

**Published:** 2021-05-26

**Authors:** Maximilian Lassi, Archana Tomar, Gemma Comas-Armangué, Rebekka Vogtmann, Dorieke J. Dijkstra, David Corujo, Raffaele Gerlini, Jonatan Darr, Fabienne Scheid, Jan Rozman, Antonio Aguilar-Pimentel, Omry Koren, Marcus Buschbeck, Helmut Fuchs, Susan Marschall, Valerie Gailus-Durner, Martin Hrabe de Angelis, Torsten Plösch, Alexandra Gellhaus, Raffaele Teperino

**Affiliations:** 1 Institute of Experimental Genetics, Helmholtz Zentrum München, German Research Center for Environmental Health Neuherberg, Germany.; 2 German Center for Diabetes Research (DZD) Neuherberg, Germany.; 3 Department of Gynecology and Obstetrics–University Hospital Essen – Essen, Germany.; 4 University of Groningen, University Medical Center Groningen, Department of Obstetrics and Gynecology, Groningen, Netherlands.; 5 Cancer and Leukemia Epigenetics and Biology Program, Josep Carreras Institute for Leukemia Research (IJC) Badalona, Spain.; 6 German Mouse Clinic, Institute of Experimental Genetics, Helmholtz Zentrum München, German Research Center for Environmental Health Neuherberg, Germany.; 7 Czech Centre for Phenogenomics, Institute of Molecular Genetics of the Czech Academy of Sciences, BIOCEV, Prumyslova 595, 252 50, Vestec, Czech Republic.; 8 Azrieli Faculty of Medicine, Bar Ilan University, Safed, Israel.; 9 Program for Predictive and Personalized Medicine of Cancer, Germans Trias i Pujol Research Institute (PMPPC-IGTP), 08916 Badalona, Spain.; 10 Chair of Experimental Genetics, School of Life Science Weihenstephan, Technische Universität München Freising, Germany.

## Abstract

Circadian rhythm synchronizes each body function with the environment and regulates physiology. Disruption of normal circadian rhythm alters organismal physiology and increases disease risk. Recent epidemiological data and studies in model organisms have shown that maternal circadian disruption is important for offspring health and adult phenotypes. Less is known about the role of paternal circadian rhythm for offspring health. Here, we disrupted circadian rhythm in male mice by night-restricted feeding and showed that paternal circadian disruption at conception is important for offspring feeding behavior, metabolic health, and oscillatory transcription. Mechanistically, our data suggest that the effect of paternal circadian disruption is not transferred to the offspring via the germ cells but initiated by corticosterone-based parental communication at conception and programmed during in utero development through a state of fetal growth restriction. These findings indicate paternal circadian health at conception as a newly identified determinant of offspring phenotypes.

## Teaser

Circadian rhythm is heritable, and paternal circadian disruption affects offspring health via stress hormones in the seminal fluid.

## INTRODUCTION

Our lives are organized around 24-hour cycles, which include light and dark phases. Organisms have developed an innate temporal program known as circadian rhythm that couples sleep-wake and fasting-feeding cycles with the light-dark cycle and allows the body to anticipate daily environmental changes and coordinate physiological activities (*1*, *2*).

In mammals, circadian rhythm is achieved through an internal clock. The hypothalamic suprachiasmatic nucleus (SCN) functions as a central oscillator, which is synchronized by the light-dark cycle (*3*). The SCN then sends humoral and neuronal signals to the peripheral circadian clocks (PCCs), present in almost all tissues and organs of the body (*3*, *4*). At the molecular level, circadian rhythms are controlled by cell autonomous transcription/translation feedback loops comprising transcriptional activators (CLOCK/BMAL1), which transcribe their own repressors (PERs/CRYs/REV-ERBs) in 24-hour cycles (*5*). While these cycles persist also in the absence of external cues, circadian rhythm responds to environmental stimuli, known as Zeitgebers (ZTs) (or time giver) (*3*). Light is the most important ZT that allows the SCN to synchronize the PCCs with the environment, but other environmental cues, such as feeding and stress, also influence circadian rhythm by modulating the interaction between the SCN and the PCCs (*3*). Desynchronization between the PCCs and the environment is known as circadian disruption (*6*).

We live in a “24-hour society” with light and food available 24/7 and increasingly tight and stressful schedules, which interfere with our normal rhythms and induce a certain degree of circadian disruption. Epidemiological studies and more controlled studies in model organisms have associated circadian disruption with increased risk to complex conditions such as metabolic, psychiatric, and oncological disorders (*7*–*11*). One open question remains on whether the consequences of circadian disruption are limited to the exposed generation or persist across generations. While a growing body of evidence suggests that maternal circadian disruption, either before or during gestation, modifies offspring phenotypes (*12*–*16*), nothing has been published (to the best of our knowledge) on the role of paternal circadian rhythm for offspring physiology and health.

Glucocorticoids (GCs) are a potent internal ZT (*17*). Their endogenous secretion in mice and humans is characterized by a prominent and robust circadian oscillation with peak anticipatory of the active phase (*18*, *19*). Partial or complete loss of this rhythm is associated to profound circadian disruption with alterations in multiple physiological functions including energy metabolism, stress response, immunity, and cognition (*20*–*22*). GCs are also essential for fetal development (*23*, *24*) and likely involved in fetal entrainment to the environment through placental-fetal communication (*12*). Exposure to high levels of GCs during pregnancy predisposes offspring to metabolic and neurological diseases later in life, most likely through impaired placental function and induction of fetal growth restriction (FGR) (*15*, *25*–*31*).

With the aim of understanding the effects of paternal circadian disruption on offspring health, we have used an established environmental model of circadian disruption in male mice—i.e., night-restricted feeding (*32*, *33*)—and analyzed offspring metabolism. Our data show that paternal circadian rhythm is important for offspring feeding behavior, glycemia, and oscillatory transcription in liver and hypothalamus. Mechanistically, our data suggest that the effects of paternal circadian disruption are transmitted to the offspring via the seminal plasma. They further highlight a potential role for corticosterone in seminal plasma in the pathogenesis of FGR and developmental programming of offspring metabolic health through transcriptional alterations in placenta and fetal tissues.

## METHODS

### Animal housing

C57BL6/J mice (males and females) were purchased from Charles River Laboratories Germany (Sandhofer Weg 7, 97633 Sulzfeld) and housed in ventilated cages at constant temperature (22° ± 1°C) with moderate humidity (50 ± 5%). The mice had unlimited access to water and, unless stated otherwise in the experiments, were fed ad libitum on a 12-hour light-dark cycle from 6:00 a.m. to 6:00 p.m. All housing protocols and animal experiments were performed according to the Institutional Animal Care and Use Committee guidelines and the European Union directive 2010/63/EU and were approved by the responsible authorities of the government of Upper Bavaria (Tierversuchsantrag no.: ROB-55.2-2532.Vet_02-17-33). All efforts were made to reduce the number of animals and minimize suffering by considerate housing and husbandry. All phenotyping procedures were examined for potential refinements. Animal welfare was assessed routinely for all mice involved.

For the generation of the founder-RF (restricted feeding) fathers (four per experimental cohort in three independent cohorts), 6-week-old C57BL6/J males were accustomed to the mouse facility for 1 week and then put on a 30-day RF schedule with access to food limited to 12 hours from 6:00 a.m. to 6:00 p.m. Control F0 males (CTR) were fed ad libitum. After 30 days, F0 males were mated to age-matched and unexposed females on ad libitum feeding schedule. To reduce the male-female interaction at conception and limit the paternal influence on the mother, we timed the mating to the day-night transition from ZT10 to ZT12 (or in the first hours of the day—from ZT1 to ZT3—when requested) and removed the fathers after positive vaginal plug inspection. To minimize variations in intrauterine and early postnatal environment, pregnant females from the same experimental group were cohoused until weaning, and litters were restricted to six pups per dam to avoid over/undernutrition during lactation.

Glucocorticoid receptor heterozygous (GR^het^) mice were provided by the group of S. Herzig at the Helmholtz Research Center in Munich and genotyped using custom primers (*GRflox1*: 5′-GGC ATG CAC ATT ACG GCC TTC T-3′/*GRflox4*: 5′-GTG TAG CAG CCA GCT TAC AGG A-3′) and an optimized polymerase chain reaction (PCR) amplification protocol [95 to 30 s ➔ 35× (55 to 30 s ➔ 72 to 60 s) ➔ 72 to 10 min]. For the generation of the GR^het^ experimental cohort, 10-week-old GR^het^ females (*n* = 6) were mated to age-matched C57BL6/J males in parallel with a C57BL6/J control mating and wild-type offspring of GR^het^ mothers (GR^WT^) phenotyped against isogenic wild-type offspring of the control mating (CTRL).

Clock mutant mice (Clock^D19^) (*34*) were provided by the group of H. Oster at the University of Lübeck. For the generation of the Clock experimental cohort, 10-week-old Clock^D19^ males (*n* = 6) were mated to age-matched C57BL6/J females in parallel with a C57BL6/J control mating and wild-type offspring of Clock^D19^ fathers (CLOCK^WT^) phenotyped against isogenic wild-type offspring of the control mating (CTRL).

### Metabolic phenotyping

#### Monitoring of weight and feeding trajectories

F1 mice were weaned at 3 weeks of age, caged in groups of four per cage with F1 mice from the same experimental group, and kept on ad libitum access to food and water. Their growth trajectories were monitored by biweekly measurement of body weight on a precision scale with two decimal places.

Food intake was measured manually every 2 months for 2 weeks by calculating the amount of food that was put in the cage minus the weight of the food pellets that were left in the cage at the end of the day. Daily food intake was indirectly calculated by dividing the net amount of food by the number of mice per cage per day.

#### Indirect calorimetry

Ten-month-old mice randomly picked from each experimental group (10 to 12 per group) were singly housed in a home cage indirect calorimetry system (TSE Systems). They were monitored over a 4-day period for 21 hours per day and fed an ad libitum chow diet. Data from the first day were discarded to reduce variation introduced by acclimatization. Data from consecutive days were treated as technical replicates, acquired, averaged, and graphed at 20-min intervals for single mouse. Food consumption was measured directly as cumulative data. To visually inspect animals’ alignment to the light-dark cycle, activity (number of beam breaks in the X and Y dimension of an ActiMot cage frame) and energy expenditure (H3, kcal/hour) data was graphed as a heatmap for single mouse and as a function of time.

#### Blood collection

Blood was collected from the tail vein of 10-month-old mice around the clock at 6-hour intervals (max. 50 μl of blood per time point), and EDTA serum was used for determination of corticosterone [enzyme-linked immunosorbent assay (ELISA), Abcam #108821, according to the manufacturer’s instructions] and insulin (ELISA, MSD #K152BZC, according to the manufacturer’s instructions). One drop of blood was directly used to measure blood glucose (using the Accu-Check Aviva glucometer and test stripes, Roche).

#### Intraperitoneal glucose tolerance test

Overnight-fasted 10-month-old mice were intraperitoneally injected with glucose (1.5 g/kg of body mass), and blood glucose excursions were monitored at 15- to 30-min intervals for 120 min (using the Accu-Check Aviva glucometer and test strips, Roche).

#### Terminal bleeding and tissue collection

Ten-month-old mice were anesthetized with a ketamine-xylazine mix and terminally bled by heart puncture at 6-hour intervals around the clock and at groups of three to four animals per time point.

Blood was collected in EDTA monovettes (Sarstedt), immediately centrifuged in a 1.5-ml Eppendorf tube to separate the plasma (10,000*g*, 4°C, 10 min), and either directly used or stored at −80°C for downstream measurements. Liver, adrenal glands, and hypothalamus were collected in RNAlater (Thermo Fisher Scientific) and either directly used for RNA extraction (TRIzol reagent, Thermo Fisher Scientific) or stored for further analyses.

### Sperm and epididymal fluid collection

Sperm and epididymal fluid were collected from 10-week-old F0 mice. Briefly, the cauda epididymis was dissected on both sides, placed in a 2-ml Eppendorf tube containing sperm motility medium, and cut into small pieces using precision scissors. Sperm was isolated using a double swim-up procedure. Briefly, after 20 min of incubation at 37°C, the sperm cloud was separated from the diluted epididymal fluid by centrifuging the mixture at room temperature for 1 min at 1000*g* and then by reincubating it at 37°C for 10 min. This gave a floating layer of sperm on top of the epididymal fluid. The sperm and epididymal fluid were separated and placed into new Eppendorf tubes. The diluted epididymal fluid was subsequently used for ELISA. Epididymal fluid is referred in the text as seminal plasma.

### In vitro fertilization and embryo transfer

In vitro fertilization (IVF) and oocyte isolation were conducted according to the standardized procedures of the INFRAFRONTIER consortium (*35*, *36*). Briefly, unexposed females were superovulated with 7.5 U of pregnant mare serum gonadotropin and 7.5 U of human chorionic gonadotropin before being sacrificed for oocyte collection. The oocytes were transferred into human tubal fluid (HTF) at 37°C and 5% CO_2_. Sperm was isolated from cauda epididymis as described before (*37*). The sperm and the oocytes were cocultured for 4 to 6 hours. Subsequently, the oocytes were transferred and incubated overnight in high-calcium HTF culture medium at 37°C and 5% CO_2_. Proper embryonic development was microscopically checked before embryo transfer to foster mothers. The two-cell embryos obtained were used for surgical bilateral oviduct transfer into foster mothers as previously described (*37*).

### RNA sequencing and data analysis

Total RNA was prepared from liver (F0-ZT0 and F1-ZT0/6/12/18), hypothalamus (F1-ZT0/6/12/18), adrenal glands (F1-ZT0/6/12/18), and placenta and fetal liver using the TRIzol reagent (Thermo Fisher Scientific) according to the manufacturer’s instructions. RNA concentration and integrity were controlled on a Bioanalyzer system (Agilent), and only RNA samples with RIN (RNA integrity number) values > 7 were used for downstream applications. Sequencing libraries were prepared by using either the Nextera Library Prep Kit (Illumina) for the liver F0 samples or the QuantSeq 3′ mRNA-Seq mRNA Library Prep Kit FWD for Illumina (Lexogen) with i7 indexes (Lexogen) according to the manufacturer’s instructions. Libraries were sequenced on an Illumina HiSeq 2500 at 75 bp (F0) or 50 bp (F1/placenta/fetal liver) single-end, with a minimum output of 40 to 50 million reads per sample. Read mapping and differential expression analysis was performed using the A.I.R. (Artificial Intelligence RNA-Seq) software from Sequentia Biotech with the following pipeline: *BBDuk* (reads trimming: http://jgi.doe.gov/data-and-tools/bbtools/bb-tools-user-guide/bbduk-guide/), *STAR* [reads mapping to the mouse genome GRCm38 (ENSEMBL): https://github.com/alexdobin/STAR], *featureCounts* (gene expression quantification: http://bioinf.wehi.edu.au/featureCounts/), and *NOISeq* [statistical analysis of differentially expressed genes (DEGs): http://bioinfo.cipf.es/noiseq/doku.php]. Compared to other methods to calculate differential expression, *NOISeq* is a data adaptive nonparametric method specifically designed to account for high variability across replicates and genes with low expression levels (*38*). Before submitting to A.I.R., raw reads obtained from Lexogen libraries were trimmed for 10 bp from the left end using *seqtk*, and further trimming and quality control of reads were done using the *bbduk.sh* script from the BBMAP software using parameters k=13; ktrim=r; useshortkmers=t; mink=5; qtrim=r; trimq=10; minlength=20; ref=Illumina TruSeq adapter sequences, A18 sequence.

Heatmap and principal components analyses (PCAs) were performed with the web application ClustVis using default parameters (*39*). Kyoto Encyclopedia of Genes and Genomes (KEGG) analysis was performed with DAVID (Database for Annotation, Visualization and Integrated and Discovery) (*40*, *41*) using default parameters. Detection and analysis of oscillating transcripts were performed using JTK_CYCLE according to the developer’s instructions (*42*). Briefly, TMM-normalized read counts were obtained for each sample. To filter the lowly expressed genes, we only included genes with an average TMM > 0.3 among replicates at all time points (ZT0 to ZT18). The following settings were used for JTK_CYCLE analysis: jtkdist (4,2), periods (4:4), jtk.init (periods, 6). A gene was observed as cycling if the adjusted *P* value was below 0.05. The genes with same lag phase were grouped together, displayed as radar plot, and annotated using the DAVID pathway analysis tool.

### cDNA synthesis and real-time PCR analysis

Total RNA was extracted using TRIzol reagent (Thermo Fisher Scientific), and 1 μg was reverse-transcribed into cDNA using commercially available kits (Applied Biosystems). Quantitative PCRs were performed on a QuantStudio 6 Real-Time PCR instrument (Thermo Fisher Scientific). Postamplification melting curve analysis was performed to check for unspecific products, and primer-only controls were included to ensure the absence of contamination. For normalization, threshold cycles (Ct values) were normalized to four different housekeeping genes (namely, *Actin*, *Gapdh*, *36b4*, and *Rplp0*). The 2-ΔΔCt (ΔCt treated–ΔCt control) method was used to calculate fold enrichments. Primers were designed using qPrimerDepot.

### Placenta histological analysis

#### Quantitative image analysis of mouse placenta

For all placental analyses, formalin-fixed and paraffin-embedded samples were sectioned parallel to the mesometrial-fetal axis at 5 μm and mounted on Superfrost Plus Slides (R. Langenbrinck, Emmendingen, Germany). Stained slides were scanned with the Aperio CS2 ScanScope slide scanner (Leica, Wetzlar, Germany) at ×20 magnification, and images were converted to TIFFs via Image Scope (version 12.3.2.8013; Leica). Scanned slides were opened (plugin “bioformats_package.jar.”) and analyzed using Fiji/ImageJ (*43*). Histological characterization of placenta, placental compartments (labyrinth and spongiotrophoblast layer), and placental glycogen cells was performed on three serial sections at three different parts (100-μm interval) in the proximity of the umbilical cord from each experimental group, respectively. Total placental area was calculated by combining measurements of labyrinth and spongiotrophoblast area; differences in placental compartment composition were measured by the ratio of labyrinth to spongiotrophoblast area as previously described (*44*, *45*). For morphometric analysis and general morphological evaluation, sections were stained with hematoxylin and eosin (H&E) and Masson-Goldner trichrome (MGT) staining. Placental glycogen stores were visualized and quantified using periodic acid–Schiff (PAS) reaction.

#### MGT staining

For morphometric analysis, sections were stained with an MGT staining kit (#3459; Carl Roth GmbH, Karlsruhe, Germany) according to the manufacturer’s protocol. Briefly, sections were deparaffinized, rehydrated, incubated for 3 min with iron hematoxylin solution, and blued for 15 min in flowing tap water, followed by 5-min Goldner’s stain I, 20-min Goldner’s stain II, and 10-min Goldner’s stain III with rinsing with 1% acetic acid solution in between and followed by a standard dehydration procedure and mounting in Xylene Mountant. MGT staining was used for morphometric characterization of muscle and collagen fibers.

#### PAS reaction staining

Placental glycogen stores were detected with the PAS reaction. Sections were deparaffinized, rehydrated, incubated for 10 min with 1% periodic acid (#HP00.1; Carl Roth), washed in tap water, incubated for 20 min with Schiff’s reagent (#X900.1; Carl Roth), and treated 3 × 2 min with sulfite water (18 ml of 10% sodium-disulfite solution + 300 ml of distilled water + 15 ml of 1 M HCl) to reduce unspecific PAS reaction. Quantification of PAS-positive area (%) within the spongiotrophoblast, as well as characterization of PAS-positive cell within the labyrinth compartment, was performed without counterstain of the nuclei.

### Statistical analysis

All figures and statistical analyses were generated using GraphPad Prism 8 (San Diego, CA). Statistical significance was tested by Student’s *t* test or analysis of variance (ANOVA) when appropriate (see figure legends for individual cases). All data were expressed as means ± SEM unless otherwise specified, and a two-tailed *P* value < 0.05 with multiple comparison correction was used to indicate statistical significance (* < 0.05, ** < 0.01, and *** < 0.001).

## RESULTS

### Thirty days of night-restricted feeding disrupt circadian rhythm in male mice

**Fig. 1:**
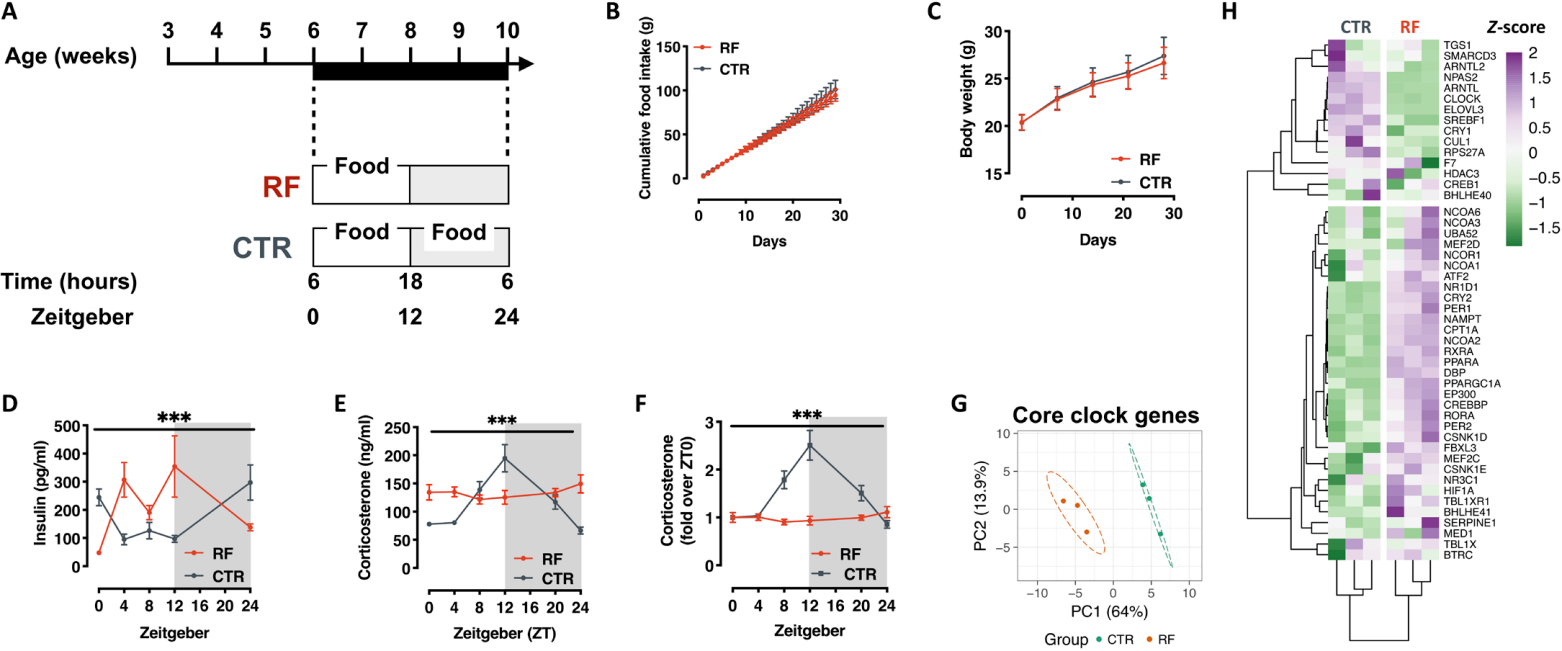
Thirty days of night-restricted feeding disrupt circadian rhythm in male mice. (**A**) Experimental design of the circadian disruption by night-RF (RF) paradigm. (**B** and **C**) Cumulative food intake (B) and body weight trajectory (C) during the 30 days of night-RF (*n* = 26). (**D**) Circulating insulin levels after 30 days of night-RF (*n* = 4 to 6). (**E** and **F**) Circulating corticosterone levels (E) and rhythmicity (F) of corticosterone secretion after 30 days of night-RF (*n* = 4 to 8). (**G**) PCA representing the variance in the expression of core clock genes in liver at Zeitgeber 0 (ZT0). (**H**) Heatmap representation of the expression of core clock genes in CTRL and RF livers at ZT0. [***P < 0.001 (two-way ANOVA mixed effect model, time × experimental group.]

In keeping with published data on the effect of time-RF on circadian rhythm (*32*, *33*), 6-week-old male C57BL6/J mice were subjected to 30 days of restricted feeding (RF; food access during the inactive phase from 6:00 a.m. to 6:00 p.m.) (Fig. 1A). After an acclimation phase of 2 to 3 days, RF mice consumed in 12 hours the same amount of food as CTR mice, which had ad libitum food access (fig. S1A). Also, the cumulative food intake over the 30-day period was not different between the groups (Fig. 1B) nor was their body weight (Fig. 1C). In keeping with a day-only feeding regimen, RF mice were hyperinsulinemic during the day (Fig. 1D) and overly circadian disrupted as shown by the 24-hour corticosterone levels, which lose its classical peak at the beginning of the night phase (Fig. 1, E and F), anticipatory of feeding and activity in nocturnal individuals (such as mice). Liver gene expression analysis at ZT0 (6:00 a.m.) revealed a profound transcriptional reprogramming with more than 8000 DEGs (more than 2000 with an absolute fold change more than 0.5; fig. S1B) annotated to key pathways for metabolic control [such as the insulin, the adenosine monophosphate–activated protein kinase (AMPK), the mammalian target of rapamycin (mTOR), and the peroxisome proliferator–activated receptor (PPAR) signaling pathways among others] (fig. S1C and table S1). Also, analysis of a set of 50 core clock genes (as reported by the REACTOME database) revealed complete transcriptional switch of the liver circadian machinery (Fig. 1, G and H), confirmed by quantitative reverse transcription–PCR expression analysis of selected candidates (such as *Cry1*, *Arntl/Bmal1*, and *Per2*) over a 24-hour period (fig.S1, D to F). Thus, 30 days of RF are sufficient to disrupt the circadian rhythm in male mice.

### Paternal circadian disruption reprograms feeding behavior, metabolic health, and oscillatory transcription in liver and hypothalamus of male offspring

**Fig. 2:**
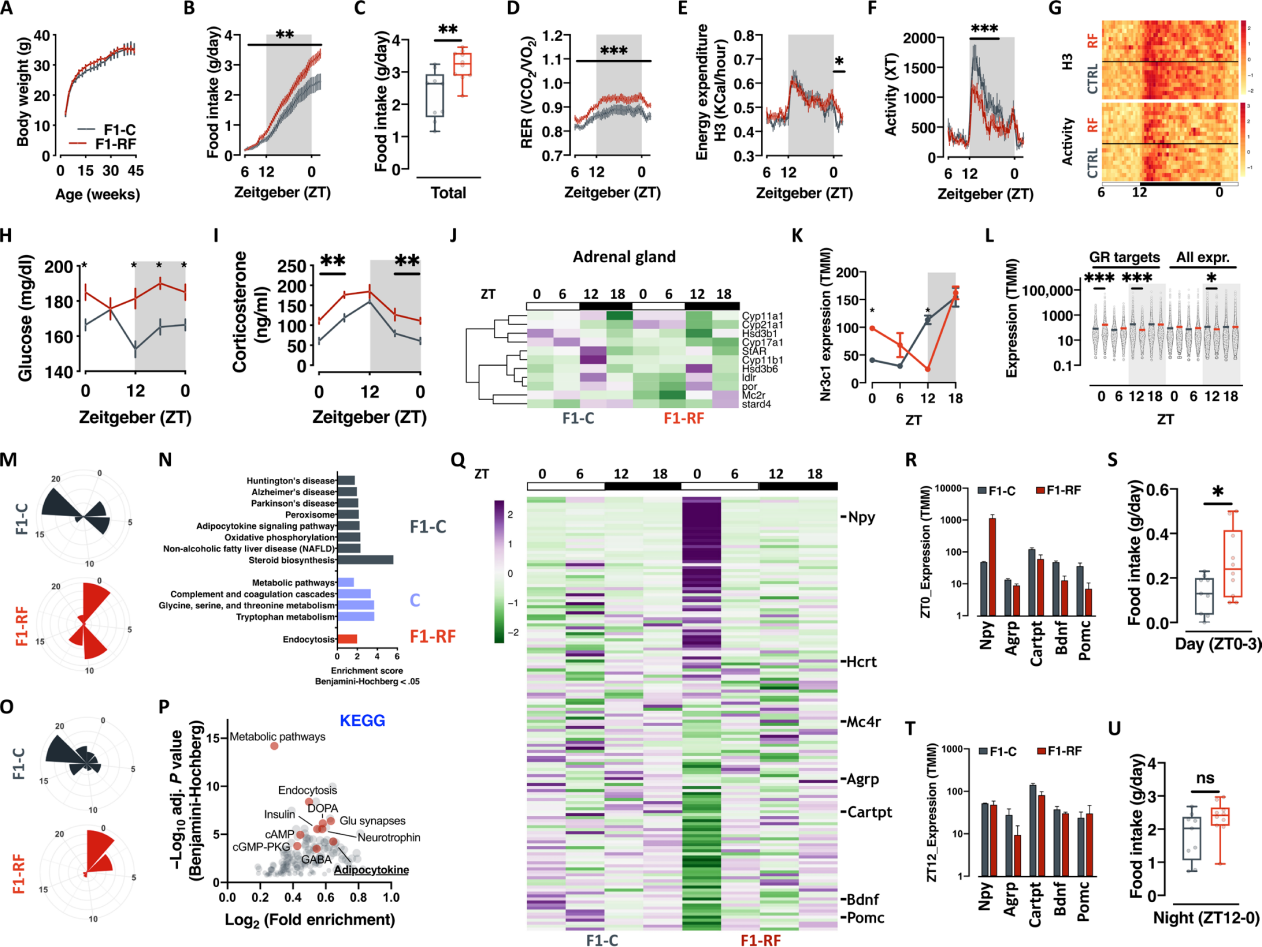
Paternal circadian disruption reprograms offspring feeding behavior, metabolic health, and oscillatory transcription in liver and hypothalamus. (**A**) Body weight trajectories. (**B** and **C**) Single animal cumulative food intake (B) and quantification of daily food intake (C). (**D** to **G**) Single animal respiratory exchange ratio (RER) (D), energy expenditure (E), locomotor activity (F), and their heatmap representation (G). (**H** and **I**) Daily oscillations in circulating glucose (H) and corticosterone (I) levels. (**J**) Heatmap of the expression of genes involved in adrenal corticosterone biosynthesis. (**K**) Liver RNA-seq–based *Nr3c1* expression. (**L**) Liver RNA-seq analysis of GR target genes from publicly available GR Chip-seq datasets. (All expr., all expressed genes in the dataset.) (**M** to **P**) JTK_CYCLE analysis of liver (M) and (N) and hypothalamus (O) and (P) RNA-seq data (M) and (O). Radar plot presenting the circadian gene expression in liver (M) and hypothalamus (O). (N) KEGG pathway analysis of oscillating genes in liver from F1-C (gray bars), F1-RF (red bar), or both (blue bars) F1 groups. (P) KEGG pathway analysis of genes differentially expressed in the hypothalamus of F1 male mice at ZT0. (**Q**) Heatmap visualization of RNA-seq–based expression of neuropeptides in F1-C and F1-RF mice. (**R** to **T**) RNA-seq–based expression of selected differentially expressed neuropeptides in the hypothalamus at ZT0 (R) and ZT12 (T) (*n* = 3 biological replicates). (**U**) Quantification of average daily food intake around ZT0 (S) and during the night phase (U). Data from F1 male mice (*n* = 10 to 12 or *n* = 3 biological replicates/ZT for RNA-seq experiments). *P < 0.05, **P < 0.01, ***P < 0.001; two-way ANOVA mixed effect model, time × experimental group or two-tailed *t* test. ns, not significant; GABA, γ-aminobutyric acid; DCPA, Dopamine or Dihidroxphenylalanine; ccMP-PKG, cyclic-GMP (Guanine Mono Phosphate)-Protein Kinase G.

Ten-week-old, RF male mice (*n* = 4 in three independent experiments) have been mated to isogenic, age-matched, and unexposed females to generate the F1 cohort. F1 males were born and developed normally, and weight monitoring from weaning to ~10 months of age showed normal growth curves and no significant difference in body weight between the F1 groups (Fig. 2A). Unexpectedly, monitoring of day and night food intake per cage in the same timeframe revealed age-dependent disruption of feeding rhythm, with significant increase in day food intake in male offspring of RF fathers (F1-RF) (fig. S2A). Indirect calorimetry-based analysis of energy metabolism in single animals (randomly selected from each independent experiment as 1 per litter, i.e., *n* = 10 to 12) confirmed overall hyperphagia in F1-RF males (Fig. 2, B and C). In keeping with the hyperphagia, F1-RF males also show higher metabolic rate [or carbohydrate oxidation rate as shown by the respiratory exchange ratio (RER)] across the 24 hours (Fig. 2D), a mild but significant increase in energy expenditure selectively around ZT0 (Fig. 2E), and remain hypoactive during the dark phase (Fig. 2F). Both energy expenditure and locomotor activity are well in phase with the day-night transition (Fig. 2G), suggesting intact light-sensing mechanism and no overt alteration of circadian rhythm. F1-RF males are also hyperglycemic (Fig. 2H) while remaining glucose tolerant as shown by normal and normally cycling circulating insulin levels (fig. S2B) and intraperitoneal glucose tolerance testing (fig. S2C).

While not overly circadian disrupted, F1-RF males are hypercorticosteronemic (Fig. 2I) with significantly dampened rhythm (fig. S2D) and out-of-phase expression of genes encoding for critical components of the corticosterone (GC) biosynthetic machinery in the adrenal glands (e.g., StAR, Cyp11a1, and Cyp11b1) (Fig. 2J and fig. S2E). In keeping with these results, liver expression of the main GR *Nr3c1* in F1-RF males shows a shifted surge from ZT12 to ZT18 and a significant over expression at ZT0 (Fig. 2K) in line with the expression of GR target genes—extracted from the analysis of publicly available GR-Chip-seq datasets (*46*)—which are globally and significantly up-regulated at ZT0 and down-regulated at ZT12 (Fig. 2L).

The effects of paternal circadian disruption are only partially detectable in female offspring. Despite no difference in feeding behavior (fig. S2F), metabolic rate (fig. S2G), and energy expenditure (fig. S2H), female offspring of circadian disrupted fathers are hypoactive (fig. S2I) and interestingly lighter (fig. S2J) compared to offspring of control fathers. Light-entrained circadian rhythm is maintained (fig. S2K), although F1-RF females show significant reduction in corticosterone levels and dampened 24-hour rhythm (fig. S2, L and M). Furthermore, despite being hyperglycemic and hyperinsulinemic during the active phase (fig. S2, N and O), they remain glucose tolerant as shown by normal glucose tolerance testing (fig. S2P). While interesting, these results are in line with recently reported findings showing profound sexual dimorphism in phenotypic traits (*47*), susceptibility to parental effects (*48*, *49*), and circadian rhythm (*50*). To reduce the overall number of animals used in the study, we decided to mechanistically follow up the feeding behavior phenotype, as the strongest and most sexual dimorphic; therefore, the rest of the results have been generated using exclusively male offspring.

These findings indicate that disruption of paternal circadian rhythm reprograms feeding behavior and metabolic health in male offspring. While not overly circadian disrupted, offspring of circadian disrupted fathers show altered GC rhythm and GR signaling with a significant shift toward day expression of GR target genes in the liver.

To understand whether the altered GC rhythm in F1-RF males is associated with a global alteration of oscillatory transcription, we performed liver RNA sequencing (RNA-seq) around the clock and used JTK_CYCLE to identify transcripts with robustly oscillating expression profiles (adj. *P* value < 0.05; table S2). We found a global transcriptional shift of approximately 6 hours in F1-RF liver (F1-C peak ~ZT18; F1-RF peak ~ZT0) (Fig. 2M), with more than 2000 transcripts losing or gaining rhythmicity (fig. S3A), and 1390 transcripts maintaining rhythmicity with an overall 6-hour shift in peak expression (fig. S3, A and B). Functional annotation of differentially oscillating transcripts (Fig. 2N) and of DEGs at ZT0 (fig. S3, C and D) shows enrichment for genes involved in glucose, lipid and amino acid metabolism, and mitochondrial oxidative capacity, suggesting altered liver adaptation to daily fast/feeding cycles in F1-RF males.

To further understand the hyperphagia and circadian phenotypes characterizing F1-RF males, we profiled hypothalamic transcription around the clock by RNA-seq and used JTK_CYCLE to identify oscillating transcripts. We observed a profound alteration of rhythmic transcription in the hypothalami of F1-RF males. Oscillating hypothalamic transcripts peak around ZT18 in F1-C and at ZT0 in F1-RF males (Fig. 2O, fig. S3E, and table S3) with a low number of genes maintaining rhythmicity (198; fig. S3F). Functional analysis of DEGs at ZT0 (fig. S3G) shows significant enrichment for lipid metabolism and adipocytokine signaling pathways, which interestingly includes key components of orexigenic and anorexigenic signaling cascades (Fig. 2, P and Q). In particular, we found strong and robust up-regulation of key orexigenic neuropeptides, such as *Npy* and *Hcrt* (precursor of the two major orexins 1 and 2) and down-regulation of anorexigenic neuropeptides, such as *Cartpt*, *Bdnf*, and *Pomc* (Fig. 2Q and table S3). These differences occur exclusively at ZT0 (Fig. 2, R and T)—when nocturnal animals stop eating—suggesting prolonged and disrupted feeding cycles in F1-RF males. Quantification of food intake in the different phases of the day revealed that—albeit overall hyperphagic (Fig. 2C)—F1-RF males consume significantly more food at the night-day transition (around ZT0) and in the first hours of the day (Fig. 2, S and U).

In keeping with the altered overall oscillatory transcription, further analysis of a set of 50 core-clock genes (as reported by the REACTOME database) in liver and hypothalamus confirmed transcriptional alterations of the clock machinery in both tissues (fig. S3, H to J). In particular, unsupervised clustering of the core-clock geneset identified three distinct clusters in both liver and hypothalamus. In liver, cluster 1 includes day-expressed genes (e.g., *Nr1d1*; fig. S3, H and I) with a delayed transcriptional peak in F1-RF males; clusters 2 and 3, instead, include genes whose transcription mostly changes at the day-night transition (e.g., *Dbp*, *Clock*, and *Per2*; fig. S3, H and I), showing either a dampened transcriptional rhythm (e.g., *Dbp* and *Clock*) or a delayed peak (e.g., *Per2*) in F1-RF males. In the hypothalamus, instead, the two major clusters are defined by genes up- or down-regulated mainly at ZT0 (cluster 1, up-regulated, including *Nr1d1*; cluster 3, down-regulated, including *Nr3c1* and *Per2*) with a third cluster (cluster 2) of unaffected genes, which includes *Clock* (fig. S3, J and K).

The intergenerational effects of paternal circadian disruption are phenocopied by paternal genetic alterations in the core-clock gene *Clock* (fig. S4A). Wild-type offspring of heterozygous Clock^D19^ mutant fathers (CLOCK^WT^) are hyperphagic despite a significantly reduced body weight (fig. S4, B to D), show a higher metabolic rate (fig. S4E), and are hypoactive with reduced overall energy expenditure (fig. S4, F and G). Also, these animals appear to have a normal light-entrained circadian rhythm (fig. S4H) while showing dampened GC rhythm (fig. S4I) and reversed glucose oscillatory behavior (fig. S4J). Thus, environmental or genetic disruption of paternal circadian rhythm reprograms feeding behavior, metabolic health, and corticosterone rhythm in male offspring, with transcriptional alterations of oscillatory genes and core clock components in liver and hypothalamus.

### The intergenerational effects of paternal circadian disruption are associated with placental and developmental signatures of FGR

Our results so far point to an important function of paternal circadian health at conception for offspring metabolic and circadian homeostasis. Although the consequences of paternal Clock mutation (fig. S4A) would suggest a direct involvement of the core clock system in the intergenerational transmission, the same system does not seem to be relevant in the male germ cells as the expression of key core-clock components (namely *Cry1*, *Arntl/Bmal1*, and *Per2*) does not oscillate in 24 hours and is not affected by the RF (fig.S4, K to M).

**Fig. 3:**
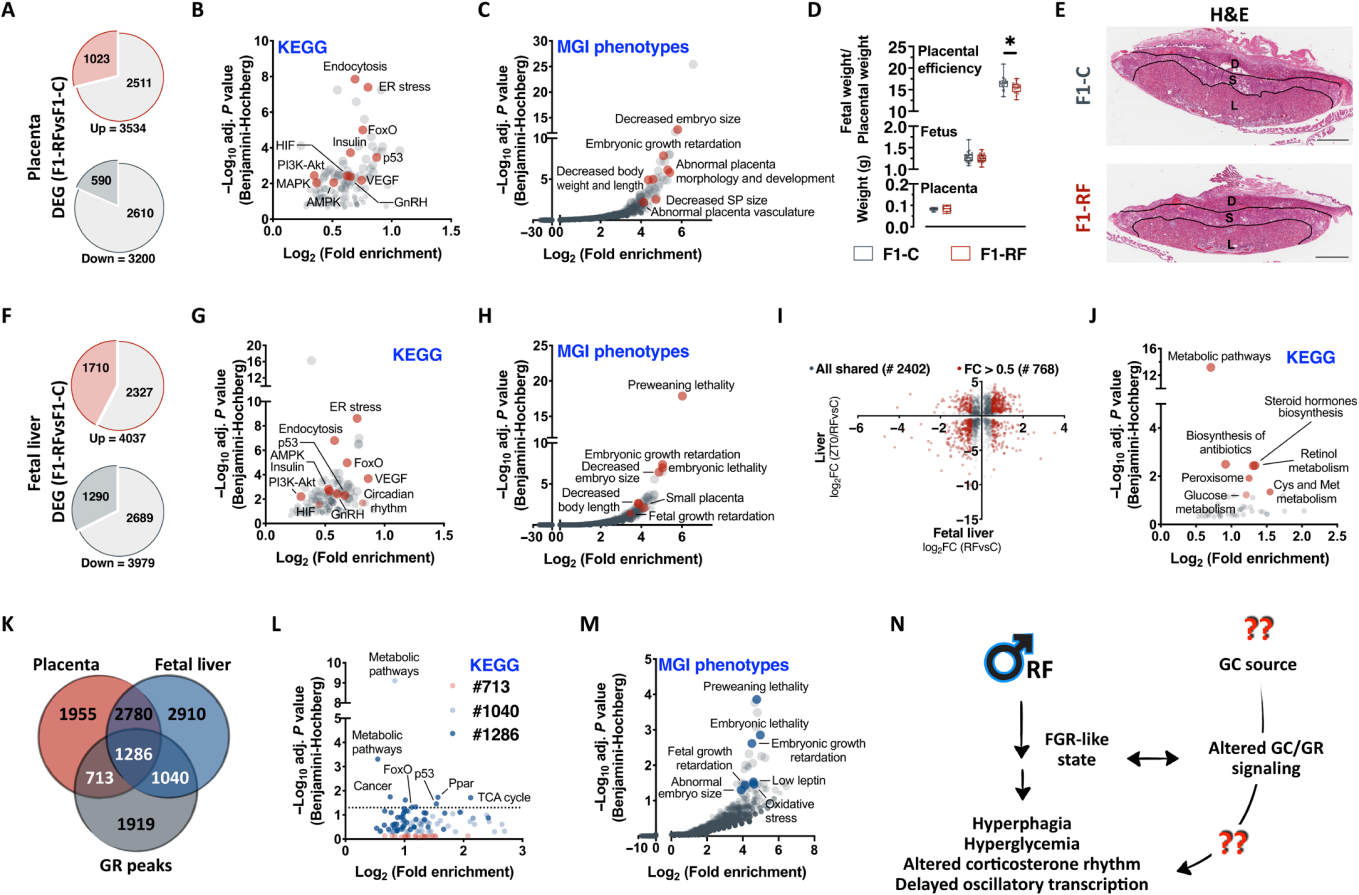
Paternal circadian disruption reprograms placenta and fetal liver transcriptomes. (**A** to **C**) Cake plot visualization of DEGs in placenta from F1-C and F1-RF male mice (A) (*n* = 4 biological replicates per group isolated between ZT2 and ZT4) and their functional annotation using KEGG pathway analysis (B) or the MGI phenotype database (C). D, Decidua; S, Spongiotrophoblast; L, Labyrinth. (**D**) Quantification of F1 placenta and fetal weight and placental efficiency at E18.5. (**E**) Representative placenta H&E staining (representative images of *n* = 4 placenta per group). (**F** to **H**) Cake plot visualization of DEGs in fetal livers from F1-C and F1-RF male mice (F) (*n* = 4 biological replicates per group) and their functional annotation using KEGG pathway analysis (G) or the MGI phenotype database (H). (**I** and **J**) Co-DEGs in fetal and adult liver (ZT0) (I) (red dots indicate genes with absolute log_2_FC > 0.5) and their functional annotation using KEGG pathway analysis (J). FC, fold change. (**K** to **M**) Venn diagram visualization of the fraction of reported GR target genes differentially expressed in the placenta and/or the fetal liver of F1-RF versus F1-C male mice (K) and their functional annotation using KEGG pathway analysis (L) or the MGI phenotype database (M). (**N**) Schematic summary of our findings reporting paternal circadian disruption associated to a signature of FGR.

On the other hand, according to the Developmental Origins of Health and Disease (*51*) hypothesis, most of the individual metabolic phenotypes are determined in utero. In particular, exposure to a hostile uterine environment has been associated—in humans and model organisms—with adult hyperphagia and late onset metabolic disorders, due to impaired placenta development and function (*52*, *53*). To understand whether the effects of paternal circadian disruption on offspring health could have been programmed in utero, we generated an independent cohort of F0 males, mated them to age-matched and unexposed females as previously described, and analyzed male placenta and fetal liver transcriptomes at Embryonic Day 18.5 (E18.5). In placenta, RNA-seq revealed more than 6500 DEGs (more than 1500 with an absolute fold change of more than 0.5) between F1-RF and F1-C male placentas (Fig. 3A and table S4), which cluster to pathways of cellular senescence (p53, insulin, and PI3K-Akt), hypoxia and oxidative stress [Forkhead Box O pathway (FoxO), hypoxia-inducible factor (HIF), and vascular endothelial growth factor (VEGF)], and energy deprivation (AMPK) and include genes critical for endocytosis and endoplasmic reticulum (ER) stress (Fig. 3B, fig. S5G, and table S4). Further interrogation of the MGI (Mouse Genome Informatics) database for mammalian gene/phenotypes associations indicated that DEGs are strongly associated to placental alterations found in mammalian models of FGR (Fig. 3C and table S4) (*54*, *55*). Despite the pronounced molecular signature of FGR, F1-RF placenta and fetuses did not show overt developmental and morphological changes apart from a mild reduction in placental efficiency (~10%, *n* = 19 to 20), measured as the ratio between fetal and placental weight (Fig. 3, D and E, and fig. S5, A to F) (*45*). The FGR-associated KEGG and MGI terms are also significantly enriched among the circa 8000 DEGs (3000 with an absolute fold change of more than 0.5) between F1-RF and F1-C fetal livers (Fig. 3, F to H; fig. S5G; and table S5), indicating that, albeit uncoupled from morphological phenotypes, the placental FGR signature is sensed by the developing fetus.

Intriguingly, we also found significant and specific enrichment of the circadian rhythm KEGG pathway in fetal liver (including core clock genes as *Per1-3*, *Cry1*, *Clock*, *Bmal1*, *Nr1d1*, and *Rora*) (fig. S5H), suggesting in utero programming of adult circadian rhythm as well. While few of the clock genes are also differentially expressed in the placenta (fig.S5H), the pathway is not significantly enriched, and the same genes are only mildly differentially expressed. Globally, placenta and fetal liver share 38% of the DEGs (fig. S5I), which increases to a maximum of 60% for DEGs belonging to the significantly enriched KEGG pathways (fig. S5I). Furthermore, comparison of fetal and adult liver (ZT0) transcriptional responses to paternal circadian disruption shows more than 2000 shared DEGs (768 with an absolute fold change of more than 0.5) (Fig. 3I and table S5), which are significantly coregulated (Fig. 3I; Spearman *r* = 0.28, *P* < 10^−4^) and cluster to glucose (*Gck, G6pc*, and *Idh1*), lipid (*Hmgcr*, *Acss2*, *Ces1d*, *Acacb*, *Ckmt1*, *Lpin3*, and *Ndufa4*), and amino acid metabolic pathways (*Ido2* and *Mthfr*) (Fig. 3J, fig. S5J, and table S5). These findings provide evidence of an FGR-like intrauterine environment as a consequence of paternal preconceptional circadian disruption and suggest it as a potential mechanistic link to the observed offspring phenotypes.

GCs and parental stress play an important role in the pathogenesis of FGR and programming of adult metabolic phenotypes (*23*, *25*, *26*). Also—relevant to our experimental question and model—GCs are important in entraining circadian rhythms (*17*, *56*, *57*) and can potentially transduce the effects of circadian disruption (*19*, *21*). GR target genes (*46*) are globally down-regulated in the placenta but not in the fetal liver of F1-RF males (fig. S5K), with more than 60% of them being differentially expressed either in the placenta or in the fetal liver (20% with an absolute fold change of more than 0.5) and a 42% (1286 of 3039 genes) overlap between the tissues (Fig. 3K). KEGG and genotype/phenotype association analysis of differentially expressed GR target genes revealed significant enrichment of pathways associated to cellular metabolism [PPAR and tricarboxylic acid (TCA) cycle; Fig. 3L and fig. S5L_1–2_] and senescence (FoxO and p53; Fig. 3L and fig. S5L_3–4_) and highlighted a genetic signature of FGR (Fig. 3M).

Thus, disruption of paternal circadian rhythm might lead to the observed offspring phenotypes through FGR and altered GC/GR signaling in the female tract (Fig. 3N). Two questions still remain to be answered: (i) What is the source of GC to the female? (ii) Is the altered GC/GR signaling sufficient to the intergenerational phenotype? (Fig. 3N).

### Corticosterone signaling at conception is important for the effects of paternal circadian disruption on offspring phenotype

**Fig. 4:**
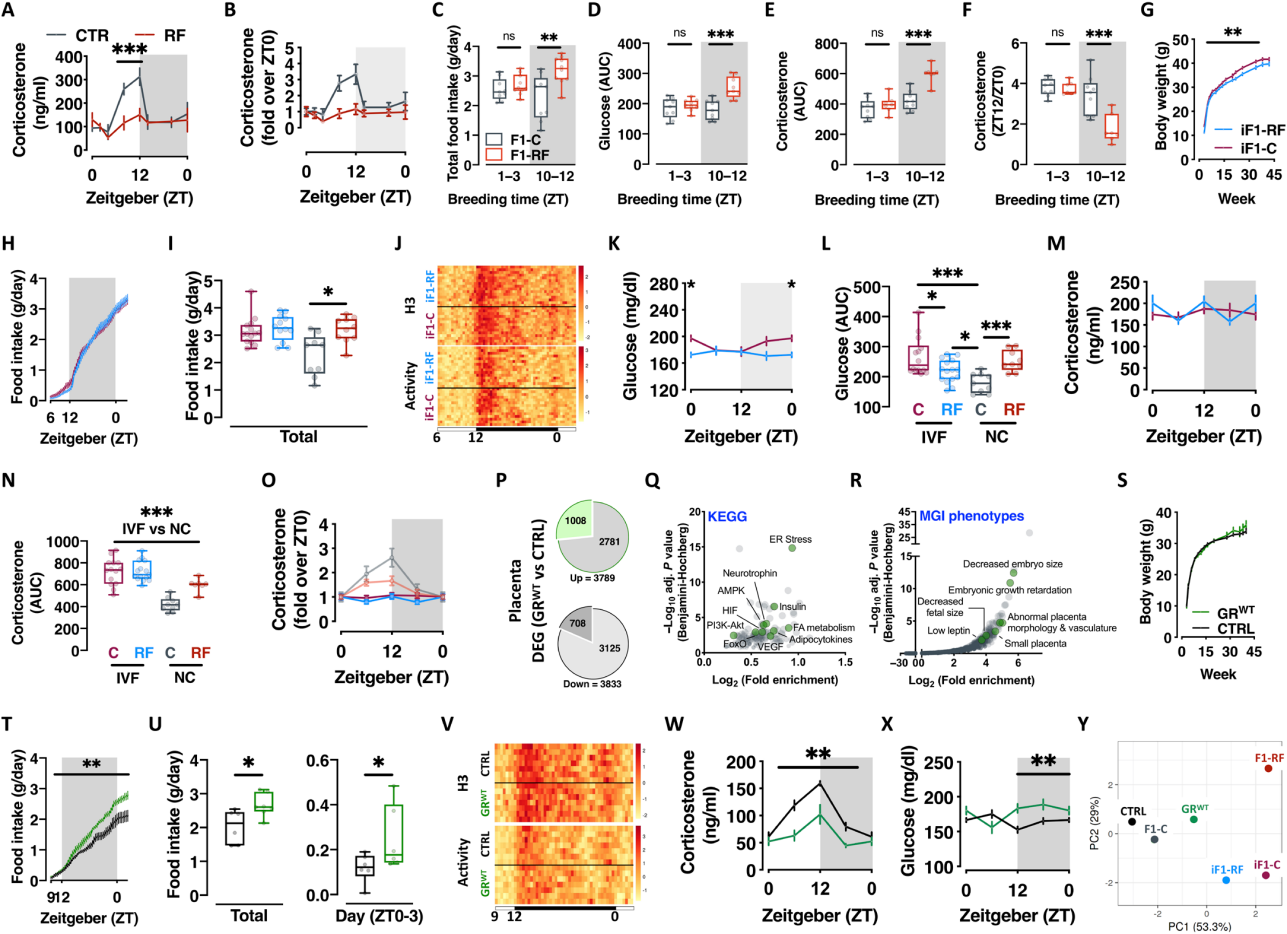
Corticosterone signaling at conception is important for the effects of paternal circadian disruption on offspring phenotype. (**A** and **B**) Corticosterone levels (A) and rhythmicity (B) in F0 mice seminal plasma (*n* = 8). (**C** to **F**) Total food intake (C), blood glucose (D), plasma corticosterone (E), and corticosterone surge at ZT12 (F) in F1-C and F1-RF males sired by parents mated at the beginning of the day (ZT1 to ZT3) or at the day-night transition (ZT10 to ZT12) (**G**) Body weight trajectories. (**H** and **I**) Single animal cumulative food intake (H) and quantification of daily total food intake compared to F1 male mice generated via natural conception (I). (**J**) Heatmap representation of single animals’ daily energy expenditure and locomotor activity. (**K** and **L**) Daily oscillations in circulating glucose levels (K) and comparison to F1 male mice generated via natural conception (L). (**M** to **O**) Daily oscillations in circulating corticosterone levels (M and O) compared to F1 male mice generated via natural conception (N). (**P** to **R**) Cake plot of DEGs in placenta (P) and their functional annotation using KEGG (Q) or the MGI phenotype database (R). (**S**) Body weight trajectories. (**T** and **U**) Single animal cumulative food intake and quantification of daily total food intake and food intake at the night-day transition (ZT0 to ZT3) (U). (**V**) Heatmap representation of single animals’ daily energy expenditure and locomotor activity. Data from IVF-generated F1 male mice (G to O) or GR^WT^ (offspring of GR^het^ mothers) and CTRL male mice (P to X) (*n* = 10 two-way ANOVA mixed effect model, time × experimental group or two-tailed *t* test; *n* = 3 biological replicates for RNA-seq experiments). (**W** and **X**) Daily oscillations in circulating corticosterone (W) and glucose (X) levels. (**Y**) PCA-based visualization of the phenotypic distance between F1 male mice from GR^het^ mothers (GR^WT^), RF fathers (F1-RF and iF1-RF generated via natural conception or IVF, respectively), and their respective controls (CTRL, F1-C, and iF1-C). AUC, area under the curve; NC, natural conception.

Seminal plasma contains trophic factors for mature spermatozoa and a battery of cytokines, hormones, and metabolites important for fertilization, implantation, placentation, and pregnancy outcome (*58*). Almost a decade ago, seminal plasma has been proposed as a potential vehicle of parental acquired information to the offspring (*59*–*62*). To test the hypothesis that seminal plasma could be the source of GC to the female at conception, we measured corticosterone concentration in seminal plasma of CTR and RF F0 males at different ZT. Corticosterone is indeed present in seminal plasma (Fig. 4A) at concentrations comparable with those in serum (see Fig. 1E for comparison), and its appearance oscillates within 24 hours with a peak at the beginning of the night phase (Fig. 4, A and B). Thirty days of night-RF severely dampened corticosterone rhythmicity in seminal plasma with a significantly less pronounced peak at the day-night transition (Fig. 4, A and B). Since our main experimental cohorts have been generated by breeding parental mice around the day-night transition (see Methods for details) and to test the hypothesis that reduced corticosterone in the seminal plasma is important for the intergenerational consequences of paternal circadian disruption, we generated a new cohort by breeding parental mice during the day (between ZT1 to ZT3) when corticosterone levels in the seminal plasma were not significantly different between CTR and RF males (Fig. 4, A and B). In keeping with our hypothesis, day breeding normalized the hyperphagia (Fig. 4C), the hyperglycemia (Fig. 4D), and the corticosterone (Fig. 4, E and F) phenotypes observed in F1-RF animals [replotted in Fig. 4 (C to F) as control reference].

To further support these findings, we used two additional approaches (fig. S6): (i) We analyzed a new cohort of F1 males generated via IVF, thereby excluding paternal factors other than germ cells (*61*) and therefore also seminal plasma and the corticosterone from the conception (fig. S6, box 1); (ii) We adopted a mouse genetics approach to reduce the GR expression at conception. We therefore used females heterozygous for the glucocorticoid receptor (GR^het^) and mated them to age-matched and unexposed males with normal and normally cycling corticosterone in the seminal plasma (fig. S6, box 2). Should our hypothesis be true, heterozygous loss of maternal GR would mimic the intrauterine environment derived from paternal circadian disruption and phenocopy its effects—at least to a certain extent and independently from genetic inheritance. As a consequence, wild-type offspring of heterozygous mothers (GR^WT^) should be phenotypically distinct from isogenic control animals (CTRL) and similar to F1-RF males.

IVF-generated animals (iF1) were born and developed normally, and weight monitoring from weaning to ~10 months of age showed normal growth curves with slight, although significant, reduction in body weight in iF1-RF males (Fig. 4G). Unexpectedly, analysis of single animal food intake revealed that iF1 animals are hyperphagic at levels similar to F1-RF males with no difference between the groups (Fig. 4, H and I), indicating that the germ cells were not sufficient to mediate the observed paternal effects. Further quantification of food intake in the different phases of the day and comparison to the same animals generated via natural conception showed that iF1 males are hyperphagic at the night-day transition and in the first hours of the day with no difference between the groups and at levels similar to F1-RF males (fig. S7A). Monitoring of energy metabolism through indirect calorimetry showed no further difference between the groups apart from a minor but significant increase in energy expenditure in iF1-C males in line with the increased body weight (fig. S7, B to D). As for F1 males generated via natural conception, iF1 males also have an intact light-sensing mechanism (Fig. 4J). Notably, despite a slight difference in basal glucose (Fig. 4K) and no difference in circulating corticosterone levels between the iF1 groups (Fig. 4M), iF1 males have blood glucose and circulating corticosterone levels comparable to F1-RF males (Fig. 4, L and N) and a dampened corticosterone diurnal rhythm (Fig. 4O).

Thus, factors in the seminal plasma are important for the effects of paternal circadian disruption on offspring phenotype. Intriguingly, by phenocopying paternal circadian disruption, the IVF experiment also suggests that seminal plasma plays a protective role. Both findings are in keeping with the reduction of corticosterone levels in seminal plasma of circadian disrupted males and therefore support our hypothesis that corticosterone might be the mediating factor.

GR^het^ females are healthy, fertile, and develop normally. They show reduced *Nr3c1* expression in the uterus (fig. S7E) and unaltered circulating corticosterone levels (fig. S7F). We mated GR^het^ females to age-matched and unexposed males and analyzed males’ placenta and fetal liver transcriptomes at E18.5, as well as the development of adult metabolic phenotypes in male wild-type offspring of GRhet mothers (GR^WT^) compared to a cohort of isogenic control animals (CTRL). Differential expression and pathway analysis of placenta RNA-seq revealed almost 8000 DEGs (almost 2000 with an absolute fold change of more than 0.5) (Fig. 4P and table S6), which cluster to pathways of cellular senescence (insulin and PI3K-Akt), hypoxia and cellular stress (HIF, FoxO, VEGF, and ER stress), and metabolic control (AMPK, fatty acid metabolism, neurotrophin, and adipocytokine) (Fig. 4Q and table S6), and highlight a signature of FGR also confirmed by interrogating the MGI database for gene/phenotypes associations (Fig. 4R and table S6). *Nr3c1* is not differentially expressed between GR^WT^ and CTRL placenta (fig. S7G). Despite the transcriptional signature of FGR in GR^WT^ placenta, morphological and developmental analysis did not show overt alterations apart from a significant reduction in placental efficiency (~25%, *n* = 6 to 12) (fig. S7, H and I). The FGR-associated KEGG and MGI terms are also significantly enriched among the circa 6000 DEGs (circa 1100 with an absolute fold change of more than 0.5) between GR^WT^ and CTRL fetal livers (fig. S7, J to L, and table S7). These findings resemble what we had previously observed in placenta and fetal livers from F1-RF males (see Fig. 3), supporting our hypothesis that interfering with corticosterone signaling in the maternal tract would phenocopy—at least to a certain extent—the effect of paternal circadian disruption on placenta and fetal liver transcriptional programming. In keeping with this, placenta and fetal livers from the two offspring cohorts share almost 60% of the DEGs (fig. S7M), which again significantly cluster to key pathways for FGR (FoxO, p53, VEGF, HIF, and PI3K-Akt), endocytosis, ER stress, and metabolic control (fatty acid metabolism, insulin, TCA cycle, and oxidative phosphorylation) (fig. S7N).

We then generated an independent cohort of GR^WT^ (from GR^het^ mothers; see fig. S6) and CTRL males and metabolically phenotyped them. GR^WT^ and CTRL males were born and developed normally, and weight monitoring from weaning to ~10 months of age showed normal growth curves and no significant difference in body weight between the groups (Fig. 4S). Indirect calorimetry-based analysis of energy metabolism in single animals showed overall hyperphagia in GR^WT^ males (Fig. 4, T and U). In keeping with the data from F1-RF males, quantification of food intake in the different phases of the day revealed that—albeit overall hyperphagic—GR^WT^ males consume significantly more food at the night-day transition (around ZT0) and in the first hours of the day (Fig. 4U). Despite no difference in metabolic rate (fig. S7O), GR^WT^ males feature a mildly but significantly higher energy expenditure (fig. S7P) in line with the mildly increased physical activity (fig. S7Q). GR^WT^ males also have an intact light-sensing mechanism (Fig. 4V) while showing dampened corticosterone rhythm (Fig. 4W) and hyperglycemia (Fig. 4X). Last, and in keeping with our original hypothesis, IVF-generated (iF1-RF and iF1-C) and GR^WT^ males are phenotypically very similar to F1-RF males (Fig. 4Y). These findings provide evidence for a role of seminal plasma corticosterone in the intergenerational consequences of paternal circadian disruption. Together, our results show that paternal circadian rhythm is important for offspring feeding behavior and metabolic health, reinforce the role of seminal plasma in acquired inheritance, and propose corticosterone as an important molecule for parental communication at conception and offspring phenotype.

## DISCUSSION

### Paternal circadian rhythm is important for offspring metabolic health

Circadian rhythm controls physiology, and disruption of normal circadian rhythm alters whole body homeostasis and predisposes to several complex conditions, including metabolic, oncological, and neurological disorders (*8*, *9*, *11*, *63*). Parental health at conception or maternal health during pregnancy are important determinants of offspring development and adult health (*64*). Among a series of environmental challenges affecting intergenerational health, recent reports have highlighted the relevance of maternal circadian rhythm for pregnancy success and offspring health in humans and model organisms (*13*–*16*). So far, instead, nothing is known about the relevance of paternal circadian rhythm. Here, we have used a validated environmental model of circadian disruption through night-RF in fathers and showed that paternal circadian rhythm is important for offspring feeding behavior, glucose control, and oscillatory transcription. As short as 30 days of night-RF in fathers is sufficient to induce hyperphagia, hyperglycemia, and disrupted corticosterone rhythm in male offspring on normal light-dark cycle and ad libitum fed. The hyperphagia is mostly contributed by food consumption at the night-day transition (around ZT0), in keeping with hypothalamic transcriptional profiles at the same time point showing deregulation of orexigenic and anorexigenic circuits. These phenotypes remind of a developmentally programmed hyperphagia and susceptibility to metabolic diseases as a consequence of in utero adverse conditions and FGR (*52*, *53*, *65*–*68*). Analysis of placenta and fetal liver reveals a transcriptional signature of FGR in offspring of circadian disrupted fathers, which is interestingly uncoupled from overt morphological and developmental alterations (placenta morphology and placental and fetal weights are normal) but associated to reduced placental efficiency and in line with the adult phenotypes. FGR is one of the reported determinants of HPA (Hypothalamic Pituitary Axis) axis activity in adulthood (*65*, *67*), and offspring of circadian disrupted fathers are constantly hypercorticosteronemic, with partially blunted diurnal corticosterone rhythm, altered expression of adrenal components of the corticosterone biosynthetic machinery, desynchronized expression of GR target genes in the liver, and a remarkable down-regulation of the GR in the hypothalamus at ZT0 [which is essential to sense circulating corticosterone and control HPA axis activity (*69*)]. Thus, paternal circadian rhythm controls offspring metabolism possibly by changing the developing environment in utero.

### Seminal fluid factors signal the status of paternal circadian rhythm

Genetic and acquired information flows from parent to offspring mostly via the gametes at conception. Lifetime environmental challenges indeed reprogram the epigenome in germ cells, and the epigenetic alterations are either transferred to the developing embryos or indirectly affect development and adult phenotypes (*64*). Almost a decade ago, also seminal plasma—the acellular part of the semen—has been proposed as a potential vehicle of parental acquired information to the offspring (*59*–*62*). Here, we have performed IVF experiments, which eliminate seminal plasma at conception and therefore its influence on the maternal tract, to isolate the role of germ cells in transmitting the effects of paternal circadian disruption to the offspring. Unexpectedly, the results of this set of experiments indicated that IVF per se phenocopied paternal circadian disruption with no difference between the experimental groups. They further suggest that not only the germ cells are not sufficient to mediate the effects of paternal circadian disruption but also that some factor(s) in the seminal plasma possibly act as a phenotypic on/off switch.

Seminal plasma contains trophic factors for mature spermatozoa and a battery of cytokines, hormones, and metabolites important for fertilization, implantation, placentation, and pregnancy outcome (*58*). Components of the seminal plasma have a high turnover rate indicating a capacity to rapidly modulate fluid composition in response to environmental challenges (*70*). Studies have shown that seminal plasma has a primary immunomodulatory role on the female tract at conception, which facilitates female receptivity and embryo implantation, therefore improving pregnancy outcome (*58*). Seminal plasma composition is altered in obese mice and men (*71*–*74*), and the absence of seminal plasma at conception modifies placenta structure and function and induces metabolic phenotypes in the offspring (*59*). Our IVF data suggest that seminal plasma is also important to control food intake, glycemia, and possibly the activity of the HPA axis, as control male mice generated via IVF are hyperphagic, hyperglycemic, and hypercorticosteronemic.

Steroids are also detectable in rodent and human seminal plasma (*73*, *74*). In humans, cortisol concentrations in the seminal plasma reach up to 60% of the serum levels (*74*). Given the role of corticosterone (the major GC in rodents) as ZT, and its response to night-RF, we also measured corticosterone in seminal plasma, which was significantly different among the experimental groups. Corticosterone concentration in the seminal plasma oscillates within 24 hours and follows the pattern observed in serum with an anticipatory peak of activity and food intake (and possibly copulatory activity) at the beginning of the night phase. In keeping with the circadian disruption observed in restricted fed animals, corticosterone rhythm is blunted also in seminal plasma, and restrictedly fed mice have significantly lower levels at the day-night transition. Conception at times when there are no differences in seminal plasma corticosterone between circadian disrupted and control mice normalizes hyperphagia, hyperglycemia, and corticosterone rhythm in male offspring.

Corticosterone has been extensively studied in model organisms and humans for its effect on pregnancy and fetal development (*23*–*26*, *28*, *75*–*78*). Altered corticosterone levels during pregnancy, and treatment with modulators of the GR, are associated with FGR and placental abnormalities with consequences for offspring health (*24*–*26*, *75*, *77*, *78*). Little is known about the role of corticosterone in seminal plasma in mice. Given that corticosterone concentrations in seminal plasma follow a 24-hour rhythm and that it responds to night-RF in mice, we hypothesized that it could be an important signaling molecule to communicate the status of paternal circadian rhythm at conception and influence offspring health by modifying the in utero environment. GR target genes in placenta—and exclusively in placenta—are significantly and globally down-regulated in male offspring of RF fathers, suggesting functional consequences to reduced corticosterone levels at conception for placentation and placental transcriptional programming. Furthermore, genetic reduction of GR expression in the female tract is sufficient to reproduce the effects of paternal circadian disruption on male offspring. Despite not carrying the genetic alteration, wild-type offspring of GR^het^ mothers develop in an environment nurtured by deficient GR signaling at conception, therefore mimicking—at least to a certain extent—what we think is happening with circadian disrupted fathers. These findings—while not constituting a formal proof—strongly propose corticosterone in the seminal plasma and corticosterone signaling in the maternal tract at conception, as a sensor of paternal circadian rhythm and determinant of offspring metabolic health.

## SUPPLEMENTARY MATERIALS

Supplementary material for this article is available at http://advances.sciencemag.org/cgi/content/full/7/22/eabg6424/DC1

## Appendix

View/request a protocol for this paper from *Bio-protocol*.
